# Prevalence of occult hepatitis B virus infection in Egypt: a systematic review with meta-analysis

**DOI:** 10.1186/s42506-023-00138-4

**Published:** 2023-07-26

**Authors:** Ahmed Azzam, Heba Khaled, Esraa S. El-kayal, Fathy A. Gad, Sarah Omar

**Affiliations:** 1 Department of Microbiology and Immunology, Faculty of Pharmacy, Helwan University, Ain Helwan, Cairo, Egypt; 2 Department of Biochemistry, Faculty of Pharmacy, Cairo University, Cairo, Egypt; 3 Biotechnology Program, Faculty of Science, Tanta University, Tanta, Egypt; 4 Faculty of Medicine, Cairo University, Cairo, Egypt; 5 Faculty of Medicine and Health Sciences, Aden University, Aden, Yemen

**Keywords:** Prevalence, Epidemiology, Occult hepatitis B, OBI, Hepatitis B virus, HBV, Meta-analysis, Genotype D, Egypt

## Abstract

**Background:**

Occult hepatitis B virus (HBV) infection (OBI) is a major public health problem. The clinical importance of OBI stems from the fact that it can be transmitted to healthy individuals at extremely low viral load levels. Additionally, immunosuppression has the potential to trigger viral replication, which can result in life-threatening liver decompensation. Despite several studies examining the prevalence of OBI, the pooled prevalence of OBI in Egypt remains unknown, particularly among blood donors and high-risk individuals, to whom intervention should be targeted.

**Methods:**

A comprehensive literature search of the following databases was conducted from inception to October 2022 using the following keywords: occult hepatitis B virus infection or occult HBV infection or OBI and Egypt in MEDLINE [PubMed], Scopus, Google Scholar, and Web of Science. The review was conducted following the Preferred Reporting Items for Systematic Reviews and Meta-Analyses (PRISMA) Statement. *I*-squared and Cochran’s *Q* were used to measure the heterogeneity between the studies, and based on the random effects model, results were reported as proportions (%) with a 95% confidence interval (CI). Analyses of subgroup analyses were conducted based on the target population. Sensitivity analyses were conducted using the leave-one-out approach to test the robustness of the results.

**Results:**

A total of 50 studies with 62 estimations of OBI were included, 19 in patients who were HBsAg-negative and anti-HBc-positive and 43 in patients who were HBsAg-negative. The highest prevalence (41%) was among multi-transfused patients according to  studies that report occult hepatitis B virus prevalence in an HBsAg-negative population, while the pooled prevalence of OBI among patients on hemodialysis, patients with chronic hepatitis C infection, patients with hepatocellular carcinoma (HCC), and patients with liver cirrhosis was 17%, 10%, 24%, and 13%, respectively. On the other hand, among studies that report OBI prevalence in HBsAg-negative and anti-HBc-positive individuals, the pooled prevalence of OBI among blood donors, patients with chronic hepatitis C infection, and patients with HCC was 12%, 15%, and 31%, respectively. Also, the majority of studies examining the genetic background of OBI have found that genotype D is the most prevalent.

**Conclusion:**

This study highlights the high prevalence in OBI among blood donors and high-risk populations in Egypt. The implementation of HBV nucleic acid amplification testing (NAT) may increase the safety of blood transfusions by excluding all HBV DNA-positive donations. However, the cost-effectiveness of these tests should be investigated.

**Supplementary Information:**

The online version contains supplementary material available at 10.1186/s42506-023-00138-4.

## Introduction

Hepatitis B virus (HBV) is a partially double-stranded DNA virus belonging to the genus *Orthohepadnavirus* and the virus family Hepadnaviridae [1]. Chronic HBV infection affects between 257 and 400 million people worldwide [2–4]. Globally, approximately 29% of cirrhosis-related deaths are attributed to HBV [5]. Hepatitis B now ranks as the 15th leading cause of global mortality worldwide [6].

According to the European Association for the Study of the Liver (EASL), HBV infection is classified into five phases: (I) HBeAg-positive chronic infection, (II) HBeAg-positive chronic hepatitis, (III) HBeAg-negative chronic infection, (IV) HBeAg-negative chronic hepatitis, and (V) HBsAg-negative phase or occult HBV infection [7]. Occult HBV infection was defined by a panel of experts as the presence of HBV DNA in the liver (with detectable or undetectable HBV DNA in the blood) in those who tested negative for HBsAg using currently available diagnostics [8]. For HBV testing, the current WHO clinical guidelines recommend an initial HBsAg test. This approach is also applicable to high-risk populations, such as people infected with the hepatitis C virus, those on hemodialysis, and those with advanced chronic liver disease of unknown etiology [9]. Unfortunately, this strategy poses the risk of overlooking OBI.

OBI can be categorized as seropositive or seronegative, defined by serum markers of HBV infection. The majority of cases are seropositive [10]. Seropositive OBI is characterized by the detection of anti-HBc antibodies with or without anti-HBs, while seronegative OBI is characterized by undetectable antibodies, both anti-HBc and anti-HBs [11]. The clinical impact of OBI includes the following: First, it plays a significant role in the progression of liver diseases, including hepatocellular carcinoma and liver cirrhosis; second, it can spread to healthy individuals even at extremely low viral load levels. Third, immunosuppressive therapies in patients with OBI may trigger HBV reactivation [12].

Globally, the overall prevalence of OBI was 0.2% (95% CI: 0.1–0.4) in HBsAg-negative blood donors [13]. The prevalence of OBI was generally higher in countries with low economic status; for instance, in Africa, OBI prevalence in HBsAg-negative blood donors was 5% (95% CI: 0.7–12.6) [13]. Regardless of the endemicity, OBI prevalence was high in high-risk groups: 5.5% (95% CI 2.9–8.7) in low-endemicity countries, 5.2% (2.5–8.6) in intermediate-endemicity countries, and 12% (3.4–24.7) in high-endemicity countries [14].

Despite several studies addressing the prevalence of OBI, the pooled prevalence of OBI in Egypt remains unknown, especially in specific subpopulations such as blood donors, those with liver-related conditions, multi-transfused patients, patients with malignancies, and healthcare workers. So, we conducted this review to completely synthesize the available data and fill this knowledge gap.

## Material and methods

### Search strategy

A comprehensive literature search of the following databases was conducted from inception to October 2022 using the following keywords: occult hepatitis B virus infection or occult HBV infection or OBI and Egypt in MEDLINE [PubMed], Scopus, Google Scholar, and Web of Science. The review was conducted following the Preferred Reporting Items for Systematic Reviews and Meta-Analyses (PRISMA) Statement and was registered in PROSPERO with registration number CRD42022368147.

### Inclusion and exclusion of studies

The following are the inclusion criteria:

1. Only primary studies (cross-sectional, case–control, or cohort studies) of participants residing in Egypt
2. Studies reporting the prevalence of occult HBV infection (HBsAg-negative and anti-HBc positive, or HBsAg-negative and anti-HBc-negative, or HBsAg-negative with unknown anti-HBc) were considered eligible regardless of the molecular detection technique of OBI
3. Studies published in English without a time limit

The following are the exclusion criteria:

1. Studies that were not conducted in Egypt or on Egyptian immigrants
2. Non-human studies
3. Full text is not available
4. Case reports, review articles, and conference abstracts

Studies were selected based on the aforementioned “inclusion and exclusion criteria” by three independent authors (H.K., S.O., and E.S.E.). All disagreements were settled by consensus.

### Data extraction

The data extraction was conducted by three investigators (A.Az., H.K., and F.A.G.) and cross-checked by (E.S.E. and S.O.). From each included study, the following was extracted: the last name of the first author, publication time, study period, age, region, population, number of participants recruited to the study, number of participants tested for HBV DNA, OBI cases, HBV-DNA detection technique, and serological criteria used to test HBV DNA. For reports that address OBI genotyping, the number of occult HBV cases undergoing genotype analysis and their distribution among different genotypes were extracted.

### Quality assessment

The quality of the included studies was checked using the “Joanna Brigg Critical Appraisal Checklist for Prevalence Studies” by two independent reviewers (S.O. and E.S.) and cross-checked by F.A.G. and A.Az.

### Data synthesis

*I*-squared and Cochran’s *Q* were used to measure the heterogeneity between the studies, and based on the random effects model, results were reported as proportions with a 95% confidence interval (CI). Analyses of the subgroups were conducted based on the target population. Sensitivity analyses were conducted using the leave-one-out approach to test the robustness of the results.

All statistical analyses were performed using Open Meta Analyst (CEBM, University of Oxford, Oxford, UK).

Publication bias testing by funnel plot and associated tests were not conducted as they do not produce reliable results for meta-analysis of proportions [15].

## Results

### Study selection

**Fig. 1:**
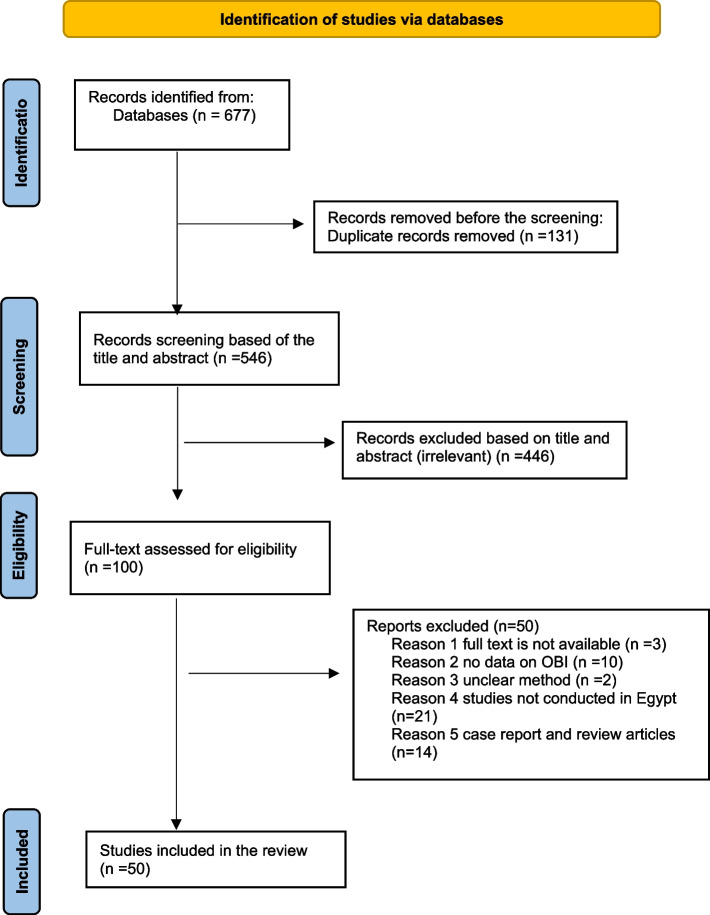
PRISMA flow chart outlining the process of article selection

A total of 677 records were identified through searching databases. There were 131 duplicates removed. The remaining 546 publications were then evaluated by title and abstract, and 446 articles were found to be irrelevant and excluded. The remaining 100 articles were evaluated for eligibility by full text, of which 50 were excluded, and a total of 50 studies reporting 62 estimates of occult HBV (Fig. 1) fulfilled our inclusion and exclusion criteria and were included in our review.

### The characteristics of the included studies

The characteristics of the studies included are shown in Table 1. A total of 62 estimations of OBI were reported by 50 studies: 19 in patients who were HBsAg-negative and anti-HBc-positive and 43 in patients who were HBsAg-negative. Among the 43 studies conducted on HBsAg-negative patients, 8 were conducted on patients undergoing hemodialysis, 4 were on multi-transfused patients, 16 were on patients with chronic hepatitis C infection, 5 were on patients with hepatocellular carcinoma (HCC), 3 were on patients with liver cirrhosis, 2 were on blood donors, 2 were on children with cancer, 2 were on healthy adults and children, and one was on high-risk children born to HBsAg-positive mothers.

**Table 1:** Characteristics of the included studies

**last name of the first author (publication year)**	**Age**^a^	**Region**	**Population**	**Study period**	**Number of participants recruited to study**	**Number of participants tested for HBV DNA**	**OBI cases**	**Prevalence (%)**	**HBV-DNA detection technique**
A: Studies that report OBI among HBsAg negative population									
Ismail (2010) [16]	20–72	Menoufia	Patients on hemodialysis	–	116	116	6	5.17	RT-PCR
Abu El Makarem (2012) [17]	48.1 ± 10.5	Minia and Assiut,	Patients on hemodialysis	2009	145	145	6	4.14	Nested PCR and RT-PCR
Saad El-Dine (2013) [18]	54 ± 11.8	Giza	Patients on hemodialysis	2009	32	32	23	71.88	Nested PCR
Mandour (2015) [19]	12–76	The Suez Canal region	Patients on hemodialysis	–	165	165	3	1.82	Nested PCR
Esmail (2016) [20]	29–63	Minia	Patients on hemodialysis	–	144	50	12	24.00	RT-PCR
Zaki (2015) [21]	26–65	Mansoura	Patients on hemodialysis	2013	96	96	18	18.75	Conventional PCR
Helaly (2015) [22]	33–63	Alexandria	Patients on hemodialysis	2012	100	100	32	32.00	RT-PCR and nested PCR
Mohamed (2020) [23]	25–78	Sharkia	Patients on hemodialysis	–	40	40	1	2.5	RT-PCR
Mohamed (2020) [24]	3–17	Minia	Multi-transfused children	2013–2014	45	45	27	60.00	Nested PCR
Shaker (2012) [25]	–	Cairo	Multi-transfused patients	2008–2010	80	80	26	32.50	RT-PCR
Said (2009) [26]	–	Cairo	Multi-transfused children		100	100	21	21.00	Nested PCR
El Sheredy (2015) [27]	–	Alexandria	Multi-transfused patients	2013	60	60	32	53.33	RT-PCR
Emara (2010) [28]	19–59	Sharkia	Patients with chronic HCV infection	–	155	155	6	3.87	RT-PCR
Daef (2017) [29]	45.0 ± 8.0	Assiut	Patients with chronic HCV infection	2015–2016	30	30	2	6.67	Conventional PCR
El-Ghitany (2013) [30]	34.88 ± 8.76	Alexandria	Patients with chronic HCV infection	–	254	254	8	3.15	Semi-nested PCR
Omar (2017) [31]	52 ± 7.2	Suez Canal	Patients with chronic HCV infection	2014–2015	200	200	17	8.50	RT-PCR
El-Sherif (2009) [32]	46.6 ± 11.7	Cairo	Patients with chronic HCV infection	2005–2006	29	29	0	0.00	RT-PCR
El-Sherif (2012) [33]	22–48	Assiut	Patients with chronic HCV infection	2010	220	50	10	20.00	RT-PCR
Mahmoud (2016) [34]	20–60	Alexandria	Patients with chronic HCV infection	–	100	100	18	18.00	Nested PCR
Selim (2011) [35]	21–60	Menoufia	Patients with chronic HCV infection	2008–2009	60	60	21	35.00	Semi-nested PCR
Naga (2019) [36]	45.2 ± 10.2	Cairo	Patients with chronic HCV infection	2004–2005	111	111	13	11.71	Conventional PCR
Thabit (2017) [37]	25–81	Assiut	Patients with chronic HCV infection	2014–2015	200	200	21	10.50	Nested PCR
Saad El-Dine (2013) [18]	58.2 ± 7.5	Giza	Patients with chronic HCV infection	2009	22	22	0	0.00	Nested PCR
El-Maraghy (2015) [38]	44.5 ± 9.8	Suez canal	Patients with chronic HCV infection	–	50	50	3	6.00	Conventional PCR
Sheneef (2012) [39]	22–59	Sohag	Patients with chronic HCV infection	2011	60	60	8	13.33	RT-PCR
Taha (2013) [40]	49.78 ± 5.04	Cairo	Patients with chronic HCV infection	2012–2013	20	20	2	10.00	Nested PCR
Mandour (2015) [19]	17–56	The Suez Canal region	Patients with chronic HCV infection	–	210	210	18	8.57	Nested PCR
El Bassuoni (2012) [41]	30–58	Menoufia	Patients with chronic HCV infection	2010	30	30	9	30	RT-PCR
Abd-Elfatah (2013) [42]	35–70	Cairo	Patients with HCC	–	100	86	20	23.26	Conventional PCR
Hassan (2011) [43]	55 ± 4.9	Cairo	Patients with HCC	–	40	40	9	22.50	Nested PCR
Daef (2017) [29]	57.1 ± 5.1	Assiut	Patients with HCC	2015–2016	30	30	3	10.00	Conventional PCR
Taha (2013) [40]	49.78 ± 5.04	Cairo	Patients with HCC	2012–2013	20	20	7	35.00	Nested PCR
El-Maksoud (2019) [44]	46.8–54.3	Mansoura	Patients with HCC	2015–2017	50	50	17	34.00	Nested PCR
El-Maksoud (2019) [44]	47–56	Mansoura	Patients with liver cirrhosis	2015–2017	50	50	13	26.00	Nested PCR
Daef (2017) [29]	52.4 ± 7.0	Assiut	Patients with liver cirrhosis	2015–2016	30	30	3	10.00	Conventional PCR
Khodeir (2018) [45]	58.5–70	Menoufia and Cairo	Patients with liver cirrhosis	–	55	44	2	4.55	RT-PCR
Raouf (2014) [46]	7 months to 16 years	Cairo	Children with cancer who have HCV positivity	2011–2013	50	50	16	32.00	Nested PCR
Raouf (2014) [46]	7 months to 16 years	Cairo	Children with cancer who were HCV negative	2011–2013	50	50	0	0.00	Nested PCR
El-Ghitany (2013) [30]	34.88 ± 8.76	Alexandria	Blood donors	–	254	254	13	5.12	Semi-nested PCR
Atef (2019) [47]		Zagazig	Blood donors	2017–2018	36,584	34,671	14	0.04	RT-PCR
ALY (2020) [48]	17–27	Assiut	University Students	2019	200	200	3	1.50	RT-PCR
Elrashidy (2014) [49]	4–17	Cairo	HB-vaccinated children and adolescent	2013–2014	170	170	0	0.00	Nested PCR
Foaud (2015) [50]	0.5–11	Cairo	Children, born to HBsAg-positive mothers	2012–2014	64	64	1	1.56	RT-PCR
B: Studies that report OBI among HBsAg negative/anti-HBc positive population									
Said (2013) [51]	–	Cairo	Blood donors	–	3167	303	52	17.16	RT-PCR
Antar (2010) [52]	21·7 ± 2·3	–	Blood donors	2007–2008	1026	80	5	6.3	RT-PCR
El-Zayadi (2008) [53]	18–54	Cairo	Blood donors	2005	760	78	9	11.5	RT-PCR
Kishk (2015) [54]	32.9 ± 6.1	–	Blood donors	–	343	44	10	22.7	RT-PCR
AwadAllah (2014) [55]	18 to 51	Zagazig	Blood donors	2013	94	94	6	6.38	RT-PCR
Mahmoud (2018) [56]	–	Assiut	Blood donors	–	300	62	9	14.5	RT-PCR
El-Sherif (2009) [32]	48.8 ± 9.6	Cairo	Patients with chronic HCV infection	2005–2006	71	71	16	22.54	RT-PCR
Kishk (2014) [57]	19–59	Ismailia	Patients with chronic HCV infection	–	162	40	3	7.50	Nested PCR
AwadAllah (2014) [55]	18–51	Zagazig	Patients with chronic HCV infection	2013	50	13	2	15.38	RT-PCR
Omar (2018) [58]	40–67	Ismailia	Patients with chronic HCV infection	–	120	52	8	15.4	RT-PCR
Hassan (2019) [59]	57.17 ± 8.67	Assiut	Patients with HCC	2016–2017	100	21	15	71.43	Conventional PCR
Ellakwa (2021) [60]	25–77	Cairo and Mansura	Patients with HCC	–	52	52	1	1.92	Nested PCR
Omar (2018) [58]	46–72	Ismailia	Patients with HCC	–	120	84	20	23.8	RT-PCR
Berbesh (2021) [61]	18–60	Cairo	Patients on hemodialysis	2017–2018	100	8	2	25.00	RT-PCR
Elbedewy (2016) [62]	24–67	Tanta	Patients on hemodialysis	2015	90	17	7	41.2	RT-PCR
Elkady (2017) [63]	36.1 ± 23.1	Sohag	Patients with hematological malignancies	2010–2011	165	54	23	42.6	RT-PCR
Elmaghloub (2017) [64]	16–64	Tanta	HCWs	2014–2015	556	132	7	5.30	Nested PCR
Abdelaziz (2019) [65]	21–55	Alexandria	HIV patients	2017	197	35	7	20.00	Semi-nested PCR
Hassan (2019) [59]	53.2 ± 9.25	Assiut	Patients with liver cirrhosis	2016–2017	100	19	11	57.89	Conventional PCR

*RT-PCR* Real-time polymerase chain reaction, *HCWs* Healthcare workers, *HCC* Hepatocellular carcinoma, *HCV* Hepatitis c virus

^a^Age is reported in years (as mean ± standard deviation or age range)

Among 19 studies on HBsAg-negative and anti-HBc-positive patients, there were 6 on blood donors, 4 on patients with chronic hepatitis C infection, 3 on patients with hepatocellular carcinoma (HCC), 2 on patients on hemodialysis, one on healthcare workers (HCWs), and one on each of the following: patients with hematological malignancies, HIV patients, and patients with liver cirrhosis.

The included studies were all published between 2010 and 2022, with only three publications dated between 2008 and 2009 [26, 32, 53].

The quality of the included studies is presented in Additional file 1: Table S1.

### Occult hepatitis B virus prevalence in HBsAg-negative patients

**Fig. 2:**
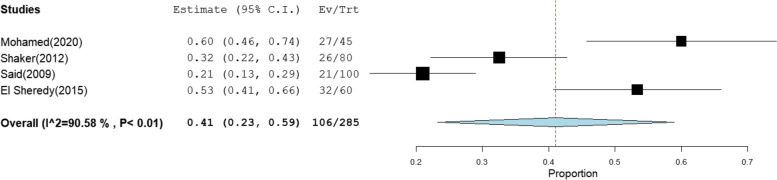
The pooled prevalence of OBI in HBsAg-negative, multi-transfused patients

**Fig. 3:**
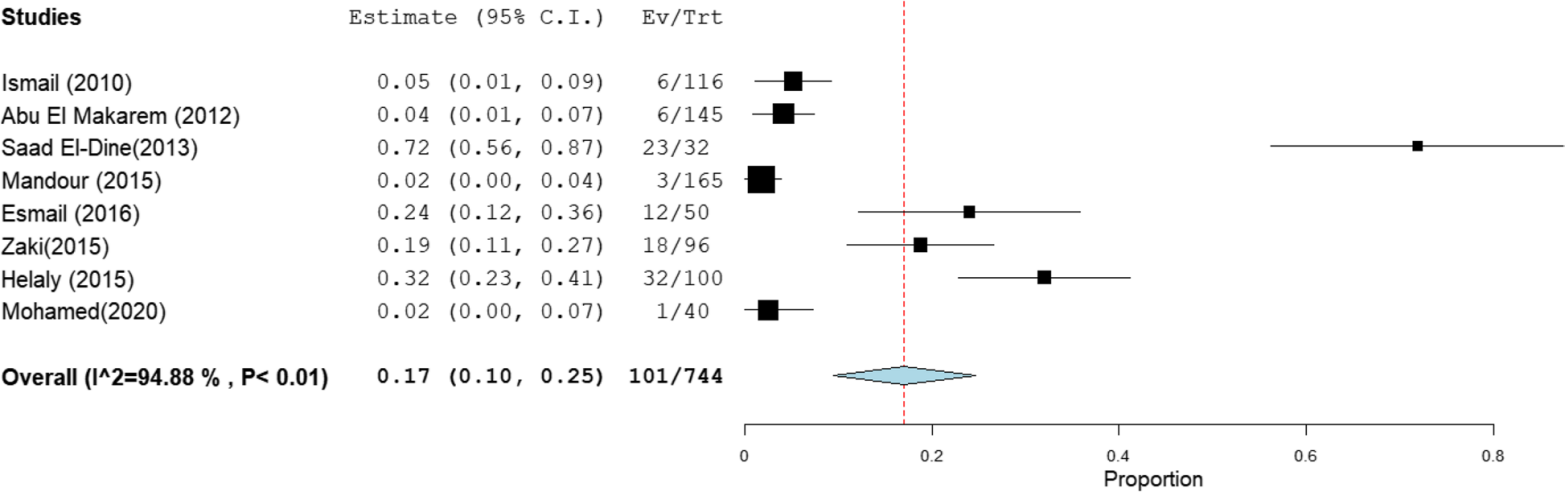
The pooled prevalence of OBI in HBsAg-negative patients on hemodialysis

**Fig. 4:**
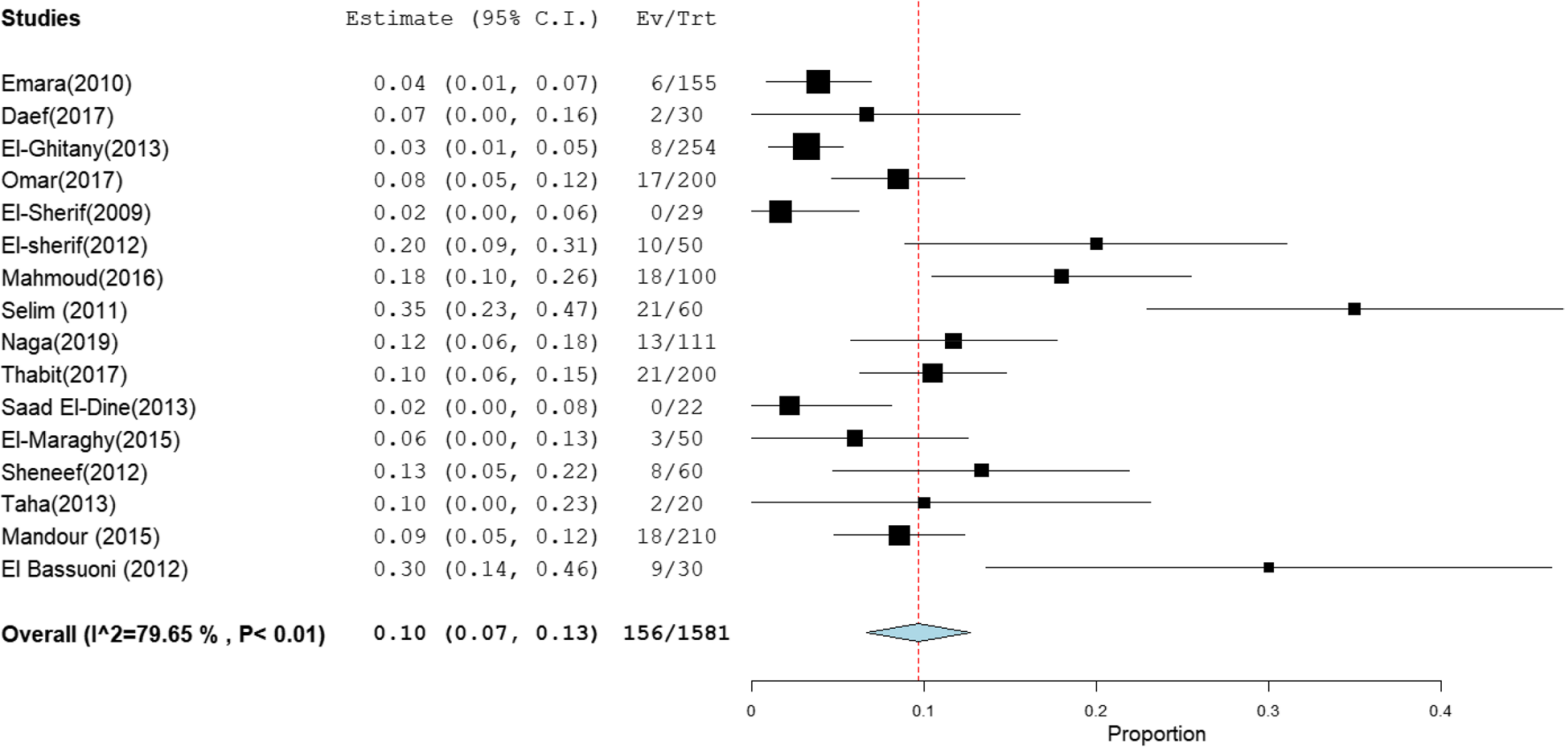
The pooled prevalence of OBI in HBsAg-negative chronic HCV-infected patients

**Fig. 5:**
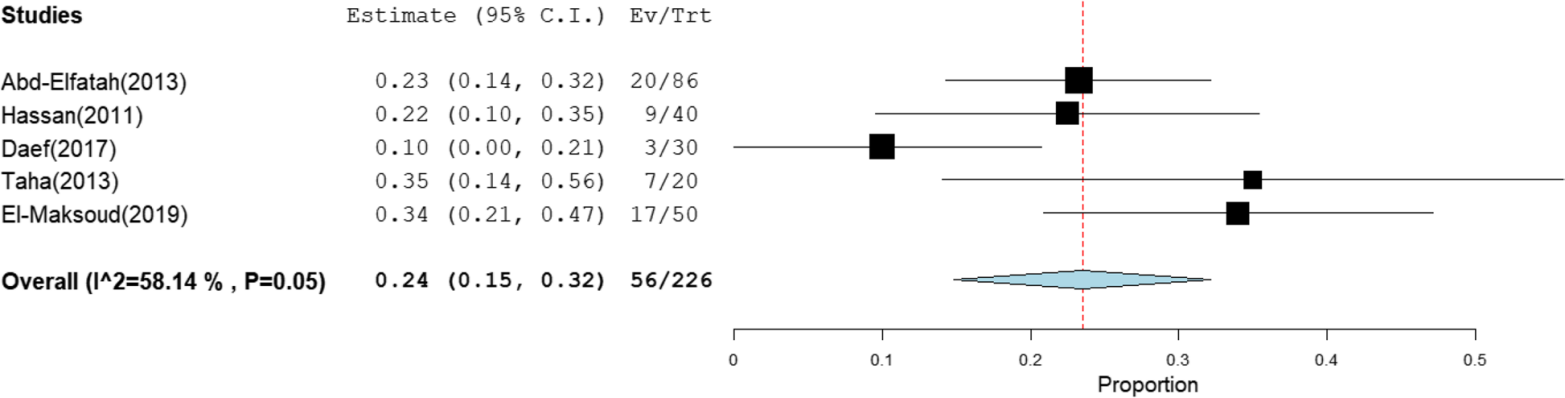
The pooled prevalence of OBI among HBsAg-negative patients with HCC

**Fig. 6:**
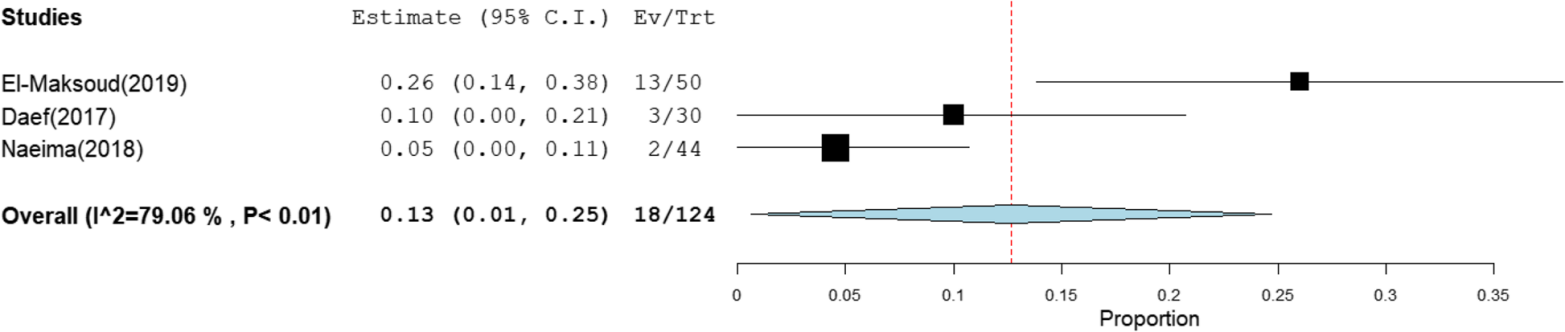
The pooled prevalence of OBI in HBsAg-negative patients with liver cirrhosis

The overall prevalence of OBI among multi-transfused patients was the highest at 41% [95% CI: 23–59] (Fig. 2).This rate was estimated from 4 studies that included 285 HBsAg-negative patients tested for HBV DNA with high heterogeneity (*I*^2^% = 90.58). The pooled prevalence of OBI among patients on hemodialysis, patients with chronic hepatitis C infection, patients with HCC, and patients with liver cirrhosis was 17% [95% CI: 10–25], 10% [95% CI: 7–13], 24% [95% CI: 15–32], and 13% [95% CI: 1–25] (Figs. 3, 4, 5, and 6), respectively. The summary of meta-analysis results for the prevalence of occult hepatitis B is presented in Table 2.

**Table 2:** Summary of meta-analysis results for the prevalence of occult hepatitis B in Egypt

Target population	No. of estimates	Participants tested for HBV DNA (*n*)	Pooled proportion (%)	95% CI	Heterogeneity		
					***I***^**2**^**% (inconsistency)**	**Cochran *Q***	***P* value**
Patients on hemodialysis	8	744	17	[10–25]	94.8	136.7	< 0.01
Multi-transfused patients	4	285	41	[23–59]	90.58	31.86	< 0.01
Patients with chronic HCV	16	1581	10	[7–13]	79.65	73.71	< 0.01
Patients with HCC	5	226	24	[15–32]	58.1	9.5	< 0.01
Patients with liver cirrhosis	3	124	13	[1–25]	79.06	9.55	< 0.01
Occult hepatitis B virus prevalence in HBsAg-negative and anti-HBc-positive							
Blood donors	6	661	12	[7–17]	72.7	18.3	< 0.01
Patients with chronic HCV	5	176	15	[8–22]	45.29	5.48	0.14
Patients with HCC	3	157	31	[1–60]	96.82	62.90	< 0.01

### Occult hepatitis B virus prevalence in HBsAg-negative and anti-HBc-positive patients

**Fig. 7:**
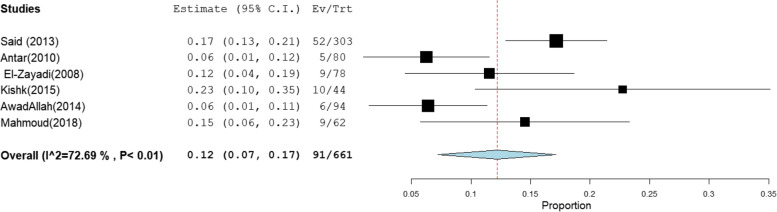
The pooled prevalence of OBI in HBsAg-negative and anti-HBc-positive blood donors

**Fig. 8:**
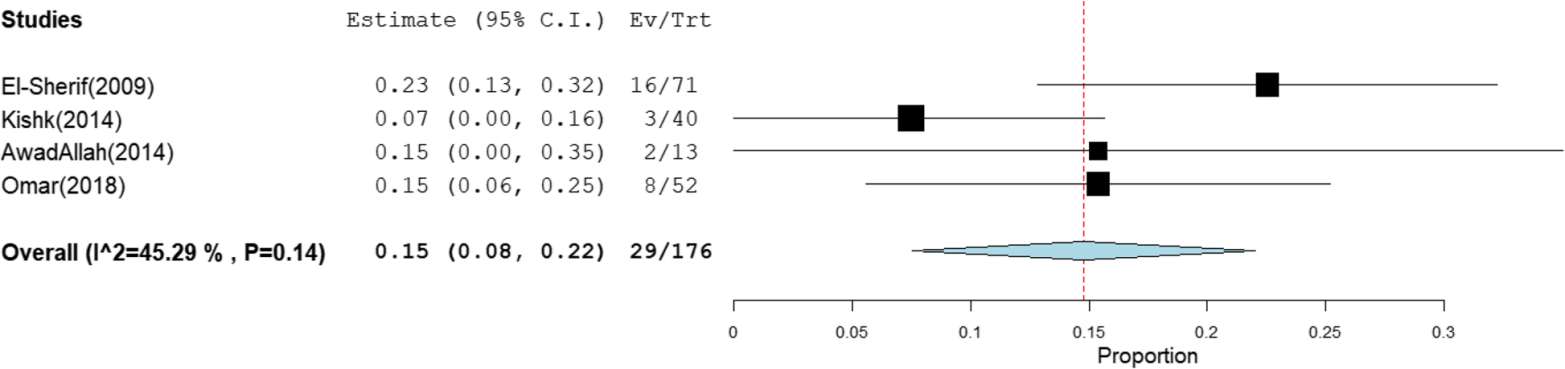
The pooled prevalence of occult hepatitis B infection in HBsAg-negative and anti-HBc-positive chronic HCV-infected patients

**Fig. 9:**
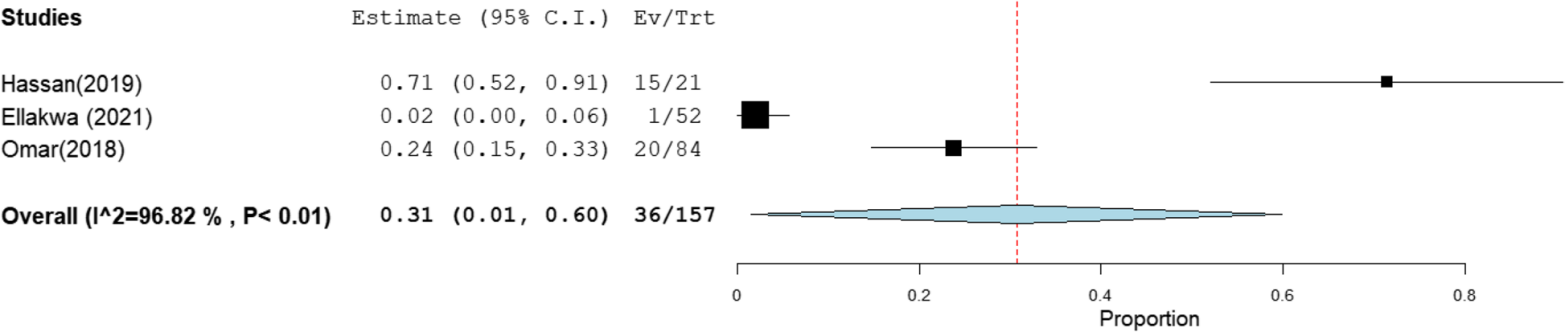
The pooled prevalence of occult hepatitis B infection in HBsAg-negative and anti-HBc-positive HCC patients

The pooled prevalence of OBI among blood donors, patients with chronic hepatitis C infection, and patients with HCC was 12% [95% CI: 7–17], 15% [95% CI: 8–22], and 31% [95% CI: 1–60] (Figs. 7, 8, and 9), respectively. The summary of meta-analysis results for the prevalence of occult hepatitis B is presented in Table 2.

### OBI genotypes distribution

**Fig. 10:**
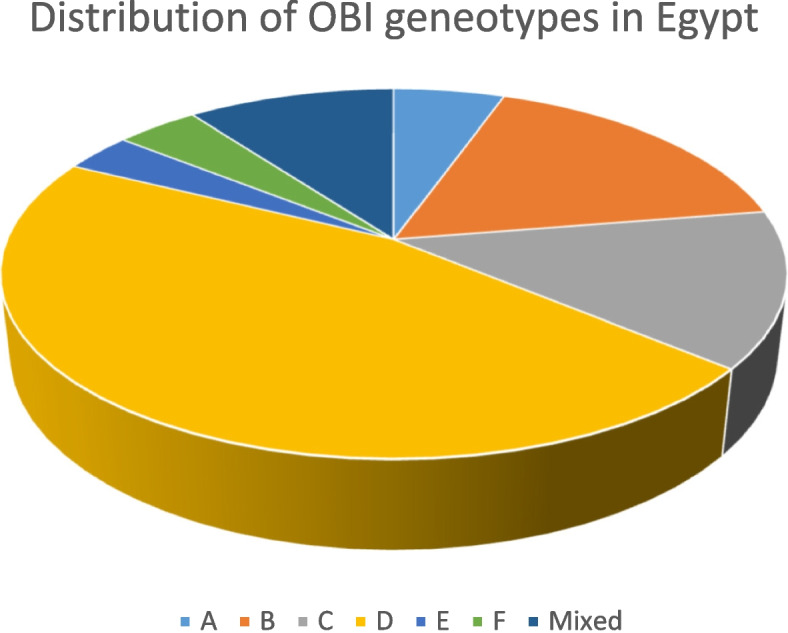
OBI genotypes distribution in Egypt

Ten reports, as shown in Table 3, addressed the genetic background of OBI, with a total number of 150 HBV genome analyses of which 68 (45.3%) were of genotype D (Fig. 10). The majority of reports (7/10) reported that genotype D predominated. However, Esmail et al., Zaki et al., and Elmaghloub et al., reported that genotypes B, E, and C were the most prevalent, [20, 21, 64] (Table 3).

**Table 3:** OBI genotype analysis among the included studies

last name of the first author (publication year)	Region	Population	Number of participants tested for HBV DNA	OBI cases (serum)	HBV genotype analysis(n)	Genotype						
						**A**	**B**	**C**	**D**	**E**	**F**	**Mixed**
Esmail (2016) [20]	Minia	Patients on hemodialysis	50	12	12	–	6	4	2	–	–	–
Hassan (2011) [43]	Cairo	Patients with HCC	40	9	25^a^	1	6	2	8			5
Taha (2013) [40]	Cairo	Patients with HCC	20	7	23^a^	2	6	3	8			4
El-Maksoud (2019) [44]	Mansoura	Patients with HCC and LC	100	30	30	–	3	3	13	2	6	3
Raouf (2014) [46]	Cairo	Cancer children who were HCV positive	50	16	16	–	–	–	–	–	–	–
Kishk (2015) [54]	–	Blood donors	44	10	10	–	–	–	–	–	–	–
Elmaghloub (2017) [64]	Tanta	HCWs	132	7	7	–	–	–	2	3		2
Kishk (2014) [57]	Ismailia	Patients with HCV	40	3	3	–	–	–	–	–	–	–
Elkady (2017) [63]	Sohag	Patients with hematological malignancies	54	23	6	–	–	–	–	–	–	–
Zaki (2015) [21]	Mansoura	Patients on hemodialysis	96	96	18	5	4	8	–	–		1
				Total	150	8	25	20	68	5	6	15

*N* Number, *LC* Liver cirrhosis

^a^The authors assess OBI in both serum and intrahepatic tissues and depend on the latter for HBV genotype analysis

### Sensitivity analysis

Sensitivity analysis using the leave-one-out approach indicated that the combined estimates are reliable and do not depend on one study (see Additional file 1: Figs. S2-S10).

## Discussion

Blood-borne hepatitis viruses are a major issue in underdeveloped nations. Egypt has one of the highest HCV prevalences in the world and is regarded as an intermediate area for HBV infection. However, the national burden of occult HBV in Egypt is still unclear. To the best of our knowledge, this is the first systematic review and meta-analysis that highlight the rise in occult HBV infection in Egypt, particularly in blood donors and high-risk populations, at which interventions should be focused.

In Egypt, HBV screening in blood banks is based only on the detection of HBsAg. However, nucleic acid amplification testing (NAT) is implemented in some large blood banks [48]. Furthermore, occult HBV infection and infection during the pre-seroconversion window period are undetectable using HBsAg assays [66]. Our study revealed that about one-tenth of blood donors who are HBsAg-negative and anti-HBc-positive have OBI in Egypt, which is consistent with a global meta-analysis among blood donors that revealed a rate of 10.0% (95% CI: 5.0–16.4) in the Eastern Mediterranean Region [13]. Oluyinka et al. suggested pre-testing Nigerian blood donors for occult HBV infections using NAT and/or anti-HBc, even if they tested negative for HBsAg, to eliminate or at least lessen the risk of HBV infection transmission [67]. However, implementing such a strategy in Egypt may not be feasible due to the high cost of nucleic acid testing as well as the need for specially trained personnel and equipment, which may not be available in many of the country’s blood banks. Furthermore, the overall sensitivity and specificity of anti-HBc as a screening tool to identify OBI were unsatisfactory (77% and 76%, respectively) [14]. So, using the anti-HBc biomarker alone to screen donated blood for occult HBV infection is not recommended, even in resource-limited countries [14].

OBI is highly prevalent among patients with end-stage renal disease (ESRD) on maintenance hemodialysis due to the frequent need for blood transfusions [68–70]. A meta-analysis of OBI in Sudan revealed a high incidence of OBI among patients on hemodialysis at 13.36%, which is lower than our findings of 17% [70], despite Sudan having a higher HBV prevalence than Egypt [4]. Based on our findings, we recommend screening for OBI in patients on hemodialysis, adherence to standard hygiene precautions, and patient isolation if OBI is detected.

Transfusion-transmitted infection (TTI) represents a real concern for transfusion services. Multi-transfused patients, such as those with thalassemia major, hemophilia, or sickle cell disease, are especially vulnerable to TTI [71]. According to the current review, multi-transfused patients have the highest OBI prevalence among the HBsAg-negative population (41%). In Iran, OBI among patients with thalassemia major was 1.16% [72]. In Palestine, no OBI cases were reported among patients with thalassemia and sickle cell anemia [73].

Occult HBV infection has frequently been identified in patients with chronic HCV infection, which has been linked to hepatocellular carcinoma and even severe liver damage [74–78]. The mechanism underlying the increased frequency of OBI in those with chronic HCV infection may include HCV molecules interfering with HBV replication, leading to OBI with reduced HBV replication [12, 79]. According to our findings, OBI was found in a significant number of HBsAg-negative patients with hepatitis C in Egypt, with a pooled prevalence of 10%, which was higher than the rate of 7.76% estimated by a similar review among Iranian HCV-positive patients. The prevalence was higher (compared with HBsAg-negative patients) at 15% among HBsAg-negative and anti-HBc-positive patients with hepatitis C infection. Several studies have demonstrated that the prevalence of OBI is not associated with the presence of anti-HCV antibodies in patients on hemodialysis [80–82].

According to Abu El Makarem et al. and Ismail et al., patients on hemodialysis with and without chronic HCV infection did not show any significant differences in the prevalence of OBI [16, 17]. Omar et al., El-Sherif et al., Mahmoud et al., and Selim et al. showed that liver aminotransferases were statistically higher in patients with dual OBI/HCV infection than those with mono-HCV infection [31, 33–35], which is consistent with other studies [83–85]. In contrast, Naga et al., Thabit et al., Taha et al., and Sheneef et al. reported that liver aminotransferases were not statistically higher in patients with dual OBI/HCV infection compared to those with mono-HCV infection [36, 37, 39, 40], which was consistent with other studies [86–89]. Therefore, the relationship between OBI and liver enzyme flare in patients with chronic HCV infection remains inconclusive. According to our findings, OBI is also frequent in patients with HCC and cirrhosis, indicating that OBI may play a role in the progression of cirrhosis and the development of HCC.

There is a paucity of studies that assessed OBI prevalence among vaccinated children. For instance, a pilot study from Taiwan revealed a 10.9% prevalence among HBV-vaccinated children [90]. Another study found that in HBsAg-negative and anti-HBc-positive subjects, OBI frequency was lower in the unvaccinated (1.7%) than in the vaccinated (4.8%) [91]. Only one study, by Elrashidy et al., evaluated the prevalence of OBI among 170 HBsAg-negative vaccinated children and adolescents with no OBI cases identified among them [49]. However, further studies with large sample sizes and long-term follow-ups are needed. There was only one study that assessed OBI in high-risk infants born to HBsAg-positive mothers by Fouad et al., demonstrating that among 64 children delivered to HBsAg-positive mothers who received HBV immunoprophylaxis (HBV vaccine and HBIG) at birth, only one case developed OBI with anti-HBc negativity [50]. Therefore, OBI may occur in infants born to HBsAg-positive mothers despite immunoprophylaxis, and being anti-HBc-negative does not rule out OBI.

### Limitation

First, the studies involved different methods of screening and kits, a resulting in variation in the sensitivity and specificity that could account for the difference in prevalence rates between the different study publication periods. Second, some studies had a small sample size. Third, there is no data about OBI prevalence in some regions. Fourth, the paucity of publications that assesse OBI in the post-vaccination era and healthy individuals. Fifth, although we stratified the included studies into subgroups to minimize the heterogeneity, there may be other sources of heterogeneity that cannot be identified. Nevertheless, our review provides crucial data on the prevalence of OBI in Egypt and highlights the high-risk populations at which intervention should be primarily targeted. Our results suggest that HBV eradication efforts should take occult HBV infection into account as a global health concern and enhance affordable access to nucleic acid testing.

## Conclusion

OBI is a major public health issue. Its clinical importance derives from the fact that OBI can spread to healthy individuals even at extremely low viral load levels. Additionally, immunosuppression has the potential to restart viral replication, which can result in life-threatening liver decompensation. The current study highlights the high prevalence of OBI among blood donors and high-risk groups, specifically patients on hemodialysis, multi-transfused patients, chronically HCV-infected patients, patients with HCC, and patients suffering from liver cirrhosis. The implementation of HBV nucleic acid amplification testing (NAT) may increase the safety of blood transfusions by excluding all HBV DNA-positive donations. However, the cost-effectiveness of these tests should be investigated. In addition, more research is required to strengthen the current evidence and describe the prevalence of OBI among vaccinated individuals.

## Supplementary Information

**Additional file 1: Fig. S1.** Supplementary Preferred Reporting Items for Systematic Reviews and Meta-analyseschecklist. Table S1. Quality assessment of the included studies. **Fig. S2.** Sensitivity analysis of the pooled prevalence of occult hepatitis B infection in HBsAg negative Hemodialysis Patients. **Fig. S3.** Sensitivity analysis of the pooled prevalence of occult hepatitis B infection in HBsAg negative multi-transfused patients. **Fig. S4.** Sensitivity analysis of the pooled prevalence of occult hepatitis B infection in HBsAg negative Chronic HCV infected patients. **Fig. S5.** Sensitivity analysis of the pooled prevalence of occult hepatitis B infection in HBsAg negative HCC patients. **Fig. S6.** Sensitivity analysis of the pooled prevalence of occult hepatitis B infection in HBsAg negative patients with liver cirrhosis. **Fig. S7.** Sensitivity analysis of the pooled prevalence of occult hepatitis B infection in HBsAg negative and anti-HBc positive blood donors. **Fig. S8.** Sensitivity analysis of the pooled prevalence of occult hepatitis B infection in HBsAg negative and anti-HBc positive HCC patients. **Fig. S9.** Sensitivity analysis of the pooled prevalence of occult hepatitis B infection in HBsAg negative and anti-HBc positive Chronic HCV infected patients. **Fig. S10.** Sensitivity analysis of the pooled prevalence of occult hepatitis B infection in HBsAg negative and anti-HBc positive HCC patients.

## Data Availability

All data generated or analyzed during this study are included in this published article [and its supplementary information file].

## Abbreviations

HBV: Hepatitis B virus
OBI: Occult hepatitis B virus infection
PRISMA: Preferred Reporting Items for Systematic Reviews and Meta-Analyses
HBsAg: Hepatitis B surface antigen
HCC: Hepatocellular carcinoma
anti-HBc: Hepatitis B core antibody
HCV: Hepatitis C virus
NAT: Nucleic acid amplification testing
EASL: European Association for the Study of the Liver
HBeAg: Hepatitis B e-antigen
Anti-HBs: Hepatitis B surface antibody
HCWs: Healthcare workers
RT-PCR: Real-time polymerase chain reaction
ESRD: End-stage renal disease
TTI: Transfusion-transmitted infection
AST: Aspartate aminotransferase
ALT: Alanine transaminase
HBIG: Hepatitis B immune globulin
