# Supplementary material for: Prevalence of occult hepatitis B virus infection in Egypt: a systematic review with meta-analysis
Source: J Egypt Public Health Assoc. 2023 Jul 26;98:13. doi: 10.1186/s42506-023-00138-4 (PMC10368600; doi:10.1186/s42506-023-00138-4)
Supplement: Supplementary file 1 — Additional file 1: Fig. S1. Supplementary Preferred Reporting Items for Systematic Reviews and Meta-analyseschecklist. Table S1. Quality assessment of the included studies. Fig. S2. Sensitivity analysis of the pooled prevalence of occult hepatitis B infection in HBsAg negative Hemodialysis Patients. Fig. S3. Sensitivity analysis of the pooled prevalence of occult hepatitis B infection in HBsAg negative multi-transfused patients. Fig. S4. Sensitivity analysis of the pooled prevalence of occult hepatitis B infection in HBsAg negative Chronic HCV infected patients. Fig. S5. Sensitivity analysis of the pooled prevalence of occult hepatitis B infection in HBsAg negative HCC patients. Fig. S6. Sensitivity analysis of the pooled prevalence of occult hepatitis B infection in HBsAg negative patients with liver cirrhosis. Fig. S7. Sensitivity analysis of the pooled prevalence of occult hepatitis B infection in HBsAg negative and anti-HBc positive blood donors. Fig. S8. Sensitivity analysis of the pooled prevalence of occult hepatitis B infection in HBsAg negative and anti-HBc positive HCC patients. Fig. S9. Sensitivity analysis of the pooled prevalence of occult hepatitis B infection in HBsAg negative and anti-HBc positive Chronic HCV infected patients. Fig. S10. Sensitivity analysis of the pooled prevalence of occult hepatitis B infection in HBsAg negative and anti-HBc positive HCC patients. [file 42506_2023_138_MOESM1_ESM.docx]

**Prevalence of Occult hepatitis B virus infection in Egypt: A systematic review with meta-analysis**

Ahmed Azzam^1^, Heba Khaled^2^, Esraa S. El-kayal^3^, Fathy A. Gad^4^, Sarah Omar^5^

^1^ Department of Microbiology and Immunology, Faculty of Pharmacy, Helwan University, Cairo, Egypt.
^2^ Department of Biochemistry, Faculty of Pharmacy, Cairo University, Cairo, Egypt.
^3^ Biotechnology program, Department of Microbiology, Faculty of Science, Tanta University, Tanta, Egypt.
^4^ Faculty of Medicine, Cairo University, Cairo, Egypt.
^5^ Faculty of Medicine and Health Sciences, Aden University, Yemen


Corresponding author: Ahmed Azzam

Department of Microbiology and Immunology, Faculty of Pharmacy, Helwan University, Cairo Egypt.

Postal address: 1 Zaki El Sisi Street, Faisal district, Giza, Egypt.

Telephone: ‎+20 0237222210

Email: ahmed.abdelkareem@pharm.helwan.edu.eg

**Fig.S1: Supplementary Preferred Reporting Items for Systematic Reviews and Meta-analyses (PRISMA) checklist**

| **Section and Topic** | **Item #** | **Checklist item** | **Location where item is reported** |
| --- | --- | --- | --- |
| **TITLE** | | |  |
| Title | 1 | Identify the report as a systematic review. | P.1 |
| **ABSTRACT** | | |  |
| Abstract | 2 | See the PRISMA 2020 for Abstracts checklist. | P.2&3 |
| **INTRODUCTION** | | |  |
| Rationale | 3 | Describe the rationale for the review in the context of existing knowledge. | P.5 |
| Objectives | 4 | Provide an explicit statement of the objective(s) or question(s) the review addresses. | P.5 |
| **METHODS** | | |  |
| Eligibility criteria | 5 | Specify the inclusion and exclusion criteria for the review and how studies were grouped for the syntheses. | P.6 |
| Information sources | 6 | Specify all databases, registers, websites, organisations, reference lists and other sources searched or consulted to identify studies. Specify the date when each source was last searched or consulted. | P.5 |
| Search strategy | 7 | Present the full search strategies for all databases, registers and websites, including any filters and limits used. | P.5 |
| Selection process | 8 | Specify the methods used to decide whether a study met the inclusion criteria of the review, including how many reviewers screened each record and each report retrieved, whether they worked independently, and if applicable, details of automation tools used in the process. | P.6 |
| Data collection process | 9 | Specify the methods used to collect data from reports, including how many reviewers collected data from each report, whether they worked independently, any processes for obtaining or confirming data from study investigators, and if applicable, details of automation tools used in the process. | P.6,7 |
| Data items | 10a | List and define all outcomes for which data were sought. Specify whether all results that were compatible with each outcome domain in each study were sought (e.g. for all measures, time points, analyses), and if not, the methods used to decide which results to collect. | P.6.7 |
|  | 10b | List and define all other variables for which data were sought (e.g. participant and intervention characteristics, funding sources). Describe any assumptions made about any missing or unclear information. | P.5-6 |
| Study risk of bias assessment | 11 | Specify the methods used to assess risk of bias in the included studies, including details of the tool(s) used, how many reviewers assessed each study and whether they worked independently, and if applicable, details of automation tools used in the process. | P.7 |
| Effect measures | 12 | Specify for each outcome the effect measure(s) (e.g. risk ratio, mean difference) used in the synthesis or presentation of results. | P.7 |
| Synthesis methods | 13a | Describe the processes used to decide which studies were eligible for each synthesis (e.g. tabulating the study intervention characteristics and comparing against the planned groups for each synthesis (item #5)). | - |
|  | 13b | Describe any methods required to prepare the data for presentation or synthesis, such as handling of missing summary statistics, or data conversions. | - |
|  | 13c | Describe any methods used to tabulate or visually display results of individual studies and syntheses. | P.7 |
|  | 13d | Describe any methods used to synthesize results and provide a rationale for the choice(s). If meta-analysis was performed, describe the model(s), method(s) to identify the presence and extent of statistical heterogeneity, and software package(s) used. | P.7 |
|  | 13e | Describe any methods used to explore possible causes of heterogeneity among study results (e.g. subgroup analysis, meta-regression). | P.7 |
|  | 13f | Describe any sensitivity analyses conducted to assess robustness of the synthesized results. | P.7 |
| Reporting bias assessment | 14 | Describe any methods used to assess risk of bias due to missing results in a synthesis (arising from reporting biases). | - |
| Certainty assessment | 15 | Describe any methods used to assess certainty (or confidence) in the body of evidence for an outcome. | - |
| **RESULTS** | | |  |
| Study selection | 16a | Describe the results of the search and selection process, from the number of records identified in the search to the number of studies included in the review, ideally using a flow diagram. | Fig.1 and P.8 |
|  | 16b | Cite studies that might appear to meet the inclusion criteria, but which were excluded, and explain why they were excluded. | P.7 and Fig.1 |
| Study characteristics | 17 | Cite each included study and present its characteristics. | Table 1 |
| Risk of bias in studies | 18 | Present assessments of risk of bias for each included study. | Table S.1 |
| Results of individual studies | 19 | For all outcomes, present, for each study: (a) summary statistics for each group (where appropriate) and (b) an effect estimate and its precision (e.g. confidence/credible interval), ideally using structured tables or plots. | Fig.(2-10) and Table 2 |
| Results of syntheses | 20a | For each synthesis, briefly summarise the characteristics and risk of bias among contributing studies. | Table 1 and Table S.1 |
|  | 20b | Present results of all statistical syntheses conducted. If meta-analysis was done, present for each the summary estimate and its precision (e.g. confidence/credible interval) and measures of statistical heterogeneity. If comparing groups, describe the direction of the effect. | Fig.(2-10) and Table 2 |
|  | 20c | Present results of all investigations of possible causes of heterogeneity among study results. | Table 2 |
|  | 20d | Present results of all sensitivity analyses conducted to assess the robustness of the synthesized results. | P.10 and Fig. S2-10 |
| Reporting biases | 21 | Present assessments of risk of bias due to missing results (arising from reporting biases) for each synthesis assessed. | - |
| Certainty of evidence | 22 | Present assessments of certainty (or confidence) in the body of evidence for each outcome assessed. | - |
| **DISCUSSION** | | |  |
| Discussion | 23a | Provide a general interpretation of the results in the context of other evidence. | P.17-19 |
|  | 23b | Discuss any limitations of the evidence included in the review. | - |
|  | 23c | Discuss any limitations of the review processes used. | - |
|  | 23d | Discuss implications of the results for practice, policy, and future research. | P.20 |
| **OTHER INFORMATION** | | |  |
| Registration and protocol | 24a | Provide registration information for the review, including register name and registration number, or state that the review was not registered. | P.5 |
|  | 24b | Indicate where the review protocol can be accessed, or state that a protocol was not prepared. | P.5 |
|  | 24c | Describe and explain any amendments to information provided at registration or in the protocol. | - |
| Support | 25 | Describe sources of financial or non-financial support for the review, and the role of the funders or sponsors in the review. | P.22 |
| Competing interests | 26 | Declare any competing interests of review authors. | P.22 |
| Availability of data, code and other materials | 27 | Report which of the following are publicly available and where they can be found: template data collection forms; data extracted from included studies; data used for all analyses; analytic code; any other materials used in the review. | P.22 |

Abbreviations: p: page.

**Table S1: Quality assessment of the included studies ***

| last name of first author (publication year) | Q1 | Q2 | Q3^β^ | Q4 | Q5 | Q6 | Q7 | Q8 | Q9 | TOTAL |
| --- | --- | --- | --- | --- | --- | --- | --- | --- | --- | --- |
| Ismail (2010)[1] | Y | N | N | Y | Y | Y | Y | Y | Y | 7 |
| Abu El Makarem (2012)[2] | Y | N | N | Y | Y | Y | Y | Y | Y | 7 |
| Saad El-Dine(2013)[3] | Y | N | N | Y | Y | Y | Y | Y | Y | 7 |
| Esmail (2016)[4] | Y | N | N | Y | Y | Y | Y | Y | Y | 7 |
| Zaki(2014)[5] | Y | N | N | Y | Y | Y | Y | Y | Y | 7 |
| Helaly (2015)[6] | Y | N | N | Y | Y | Y | Y | Y | Y | 7 |
| Mohamed(2020)[7] | Y | Y | N | Y | Y | Y | Y | Y | Y | 8 |
| Mohamed(2020)[8] | Y | N | N | Y | Y | Y | Y | Y | Y | 7 |
| Shaker(2012)[9] | Y | N | N | Y | Y | Y | Y | Y | Y | 7 |
| Said(2009)[10] | Y | N | N | Y | Y | Y | Y | Y | Y | 7 |
| El Sheredy(2015)[11] | Y | N | N | Y | Y | Y | Y | Y | Y | 7 |
| Emara(2010)[12] | Y | N | N | Y | Y | Y | Y | Y | Y | 7 |
| Omar(2017)[13] | Y | U | N | Y | Y | Y | Y | Y | Y | 7 |
| El-Sherif(2009)[14] | Y | U | N | Y | Y | Y | Y | Y | Y | 7 |
| El-sherif(2012)[15] | Y | U | N | Y | Y | Y | Y | Y | Y | 7 |
| Mahmoud(2016)[16] | Y | N | N | Y | Y | Y | Y | Y | Y | 7 |
| Selim (2011)[17] | Y | N | N | Y | Y | Y | Y | Y | Y | 7 |
| Naga(2019)[18] | Y | N | N | Y | Y | Y | Y | Y | Y | 7 |
| Thabit(2017)[19] | Y | N | N | Y | Y | Y | Y | Y | Y | 7 |
| El Bassuoni (2012)[20] | Y | N | N | Y | Y | Y | Y | Y | Y | 7 |
| El-Maraghy(2015)[21] | Y | N | N | Y | Y | Y | Y | Y | Y | 7 |
| Sheneef(2012)[22] | Y | N | N | Y | Y | Y | Y | Y | Y | 7 |
| Mandour (2015)[23] | Y | U | N | Y | Y | Y | Y | Y | Y | 7 |
| Hassan(2011)[24] | Y | U | N | Y | Y | Y | Y | Y | Y | 7 |
| Abd-Elfatah(2013)[25] | Y | U | N | Y | Y | Y | Y | Y | Y | 7 |
| Daef(2017)[26] | Y | U | N | Y | Y | Y | Y | Y | Y | 7 |
| Taha(2013)[27] | Y | Y | N | Y | Y | Y | Y | Y | Y | 8 |
| El-Maksoud(2019)[28] | Y | Y | N | Y | Y | Y | Y | Y | Y | 8 |
| Khodeir(2018)[29] | Y | Y | N | Y | Y | Y | Y | Y | Y | 8 |
| Raouf(2014)[30] | Y | Y | Y | Y | Y | Y | Y | Y | Y | 9 |
| El-Ghitany(2013)[31] | Y | Y | Y | Y | Y | Y | Y | Y | Y | 9 |
| Atef (2019)[32] | Y | N | Y | Y | Y | Y | Y | Y | Y | 8 |
| Abdel-Azeem(2020)[33] | Y | Y | N | Y | Y | Y | Y | Y | Y | 8 |
| Foaud(2015)[34] | Y | U | N | Y | Y | Y | Y | Y | Y | 7 |
| Elrashidy (2014)[35] | Y | U | N | Y | Y | Y | Y | Y | Y | 7 |
| Said (2013)[36] | Y | U | Y | Y | Y | Y | Y | Y | Y | 8 |
| Antar(2010)[37] | Y | U | Y | Y | Y | Y | Y | Y | Y | 8 |
| El-Zayadi(2008)[38] | Y | U | Y | Y | Y | Y | Y | Y | Y | 8 |
| Mahmoud(2018) [39] | Y | N | N | Y | Y | Y | Y | Y | Y | 7 |
| Kishk(2015)[40] | Y | Y | Y | Y | Y | Y | Y | Y | Y | 9 |
| Abdelaziz (2019)[41] | Y | N | N | N | Y | Y | Y | Y | Y | 6 |
| Elmaghloub(2017)[42] | Y | U | N | Y | Y | Y | Y | Y | Y | 7 |
| Kishk(2014)[43] | Y | N | N | Y | Y | Y | Y | Y | Y | 7 |
| AwadAllah(2014)[44] | Y | U | N | Y | Y | Y | Y | Y | Y | 8 |
| Omar(2018)[45] | Y | N | N | Y | Y | Y | Y | Y | Y | 7 |
| Hassan(2019)[46] | Y | U | N | Y | Y | Y | Y | Y | Y | 7 |
| Ellakwa (2021)[47] | Y | N | N | Y | Y | Y | Y | Y | Y | 7 |
| Berbesh(2021)[48] | Y | N | Y | Y | Y | Y | Y | Y | Y | 8 |
| Elbedewy(2016)[49] | Y | U | N | Y | Y | Y | Y | Y | Y | 7 |
| Elkady (2017)[50] | Y | N | N | Y | Y | Y | Y | Y | Y | 7 |

* The quality assessment was based on Joana brigs Critical Appraisal tool for prevalence studies that consist of 9 questions
Q1.Was the sample frame appropriate to address the target population?, Q2.Were study participants sampled in an appropriate way?, Q3.Was the sample size adequate?, Q4.Were the study subjects and the setting described in detail?, Q5.Was the data analysis conducted with sufficient coverage of the identified sample? , Q6.Were valid methods used for the identification of the condition? Q7.Was the condition measured in a standard, reliable way for all participants, Q8.Was there appropriate statistical analysis? And Q9.Was the response rate adequate, and if not, was the low response rate managed appropriately?

β: The sample size is considered adequate if it is more than 250 based on the following calculation: n = Z^2^P(1-P)/d2[51]

Where:

n= sample size

Z = Z statistic for a level of 95% confidence (1.96).

P = Expected prevalence or proportion (We estimate the expected prevalence or proportion to be between 0.06-0.2.)

d = precision (d=0.05)

**Fig.S2**


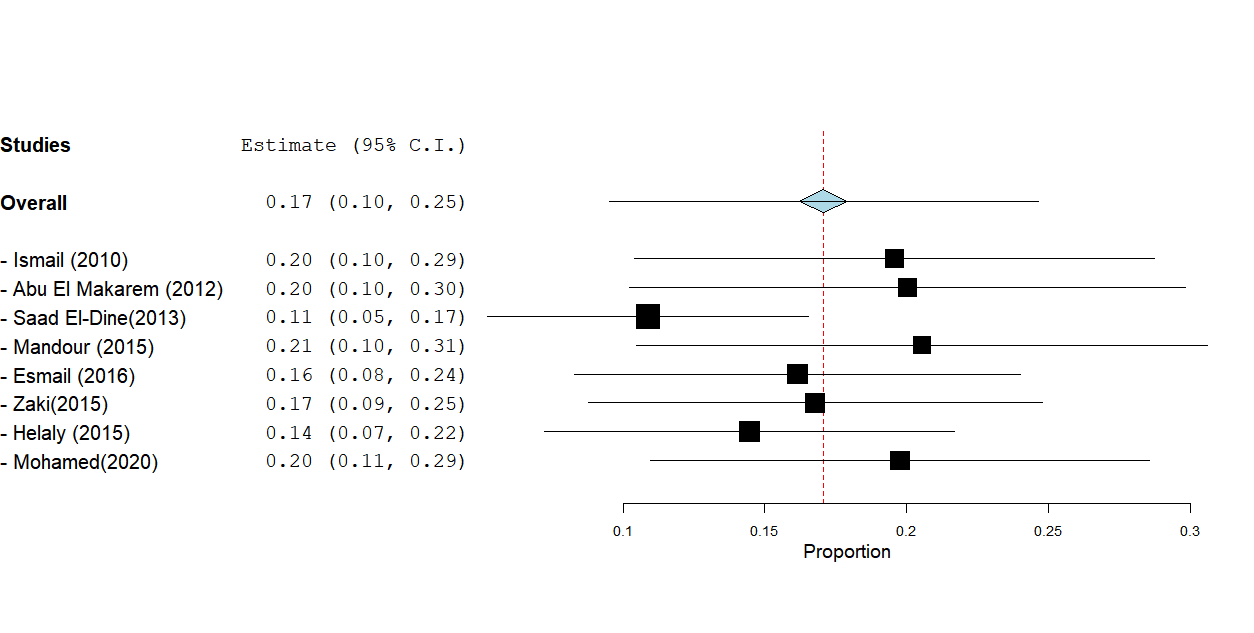


Sensitivity analysis of the pooled prevalence of occult hepatitis B infection in HBsAg negative Hemodialysis Patients.

**Fig.S3**


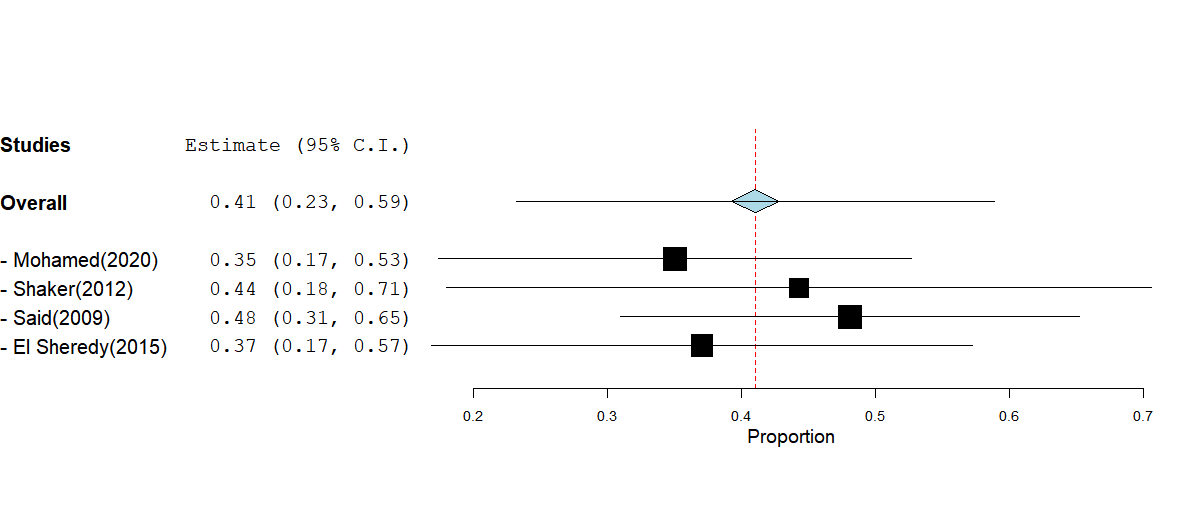


Sensitivity analysis of the pooled prevalence of occult hepatitis B infection in HBsAg negative multi-transfused patients.


**Fig.S4**
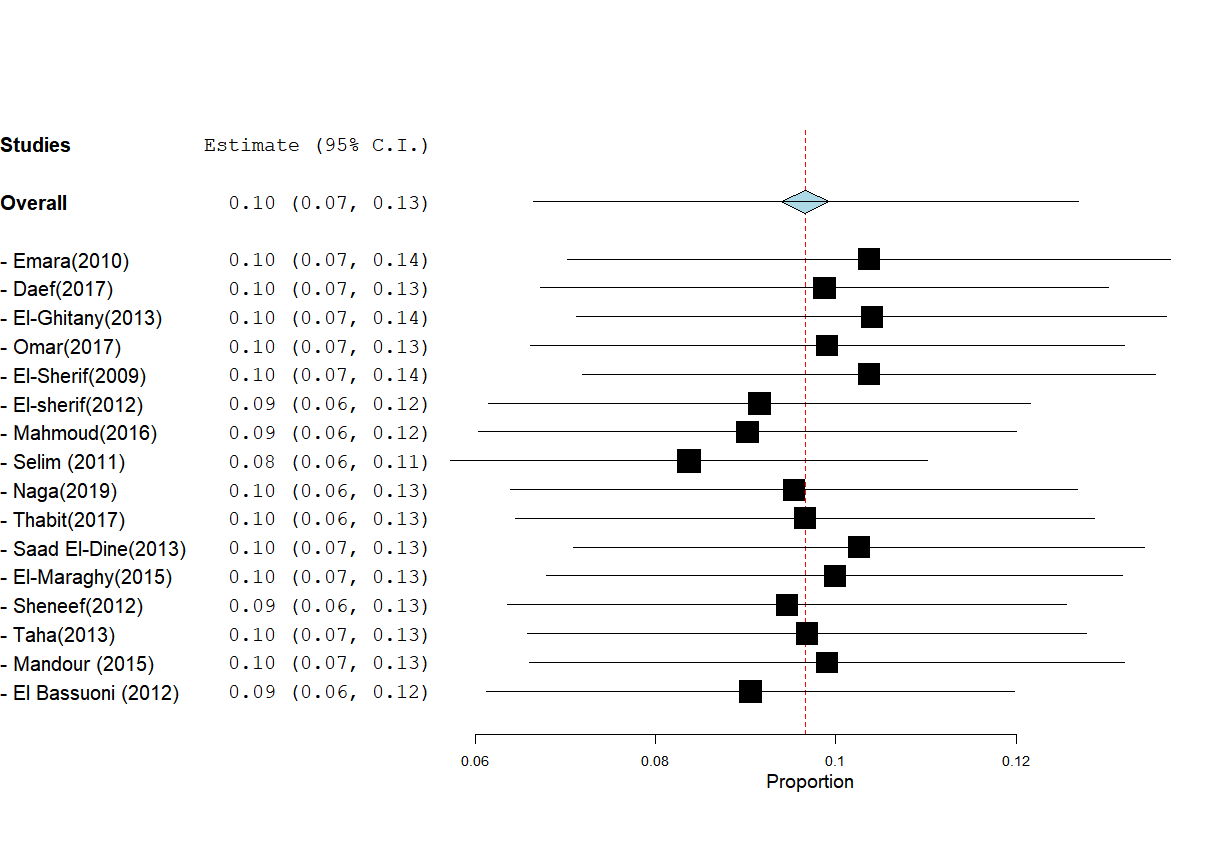


Sensitivity analysis of the pooled prevalence of occult hepatitis B infection in HBsAg negative Chronic HCV infected patients.


**Fig.S5**


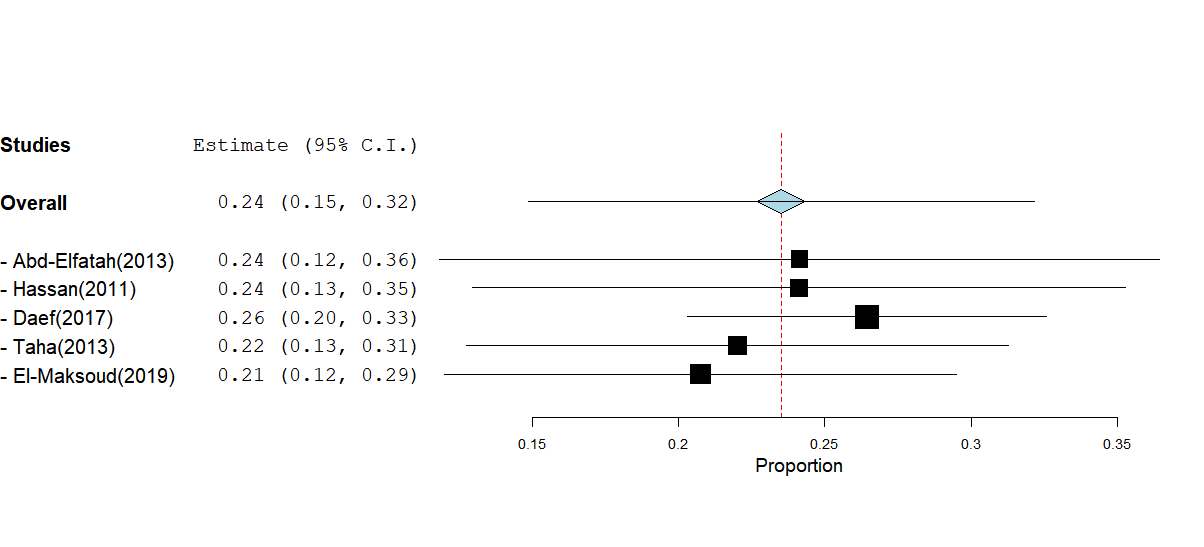


Sensitivity analysis of the pooled prevalence of occult hepatitis B infection in HBsAg negative HCC patients.


**Fig.S6**


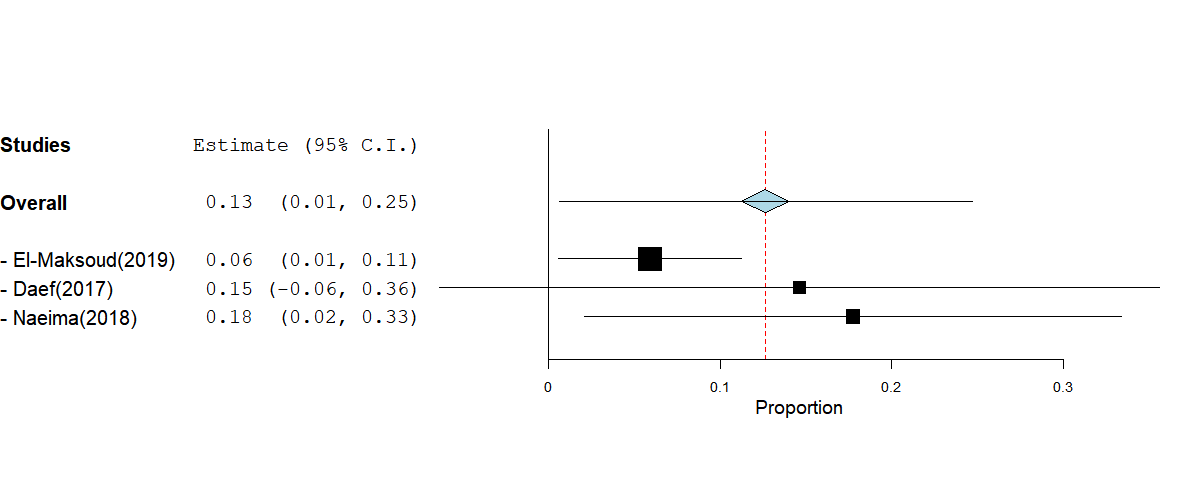


Sensitivity analysis of the pooled prevalence of occult hepatitis B infection in HBsAg negative patients with liver cirrhosis.

**Fig.S7**


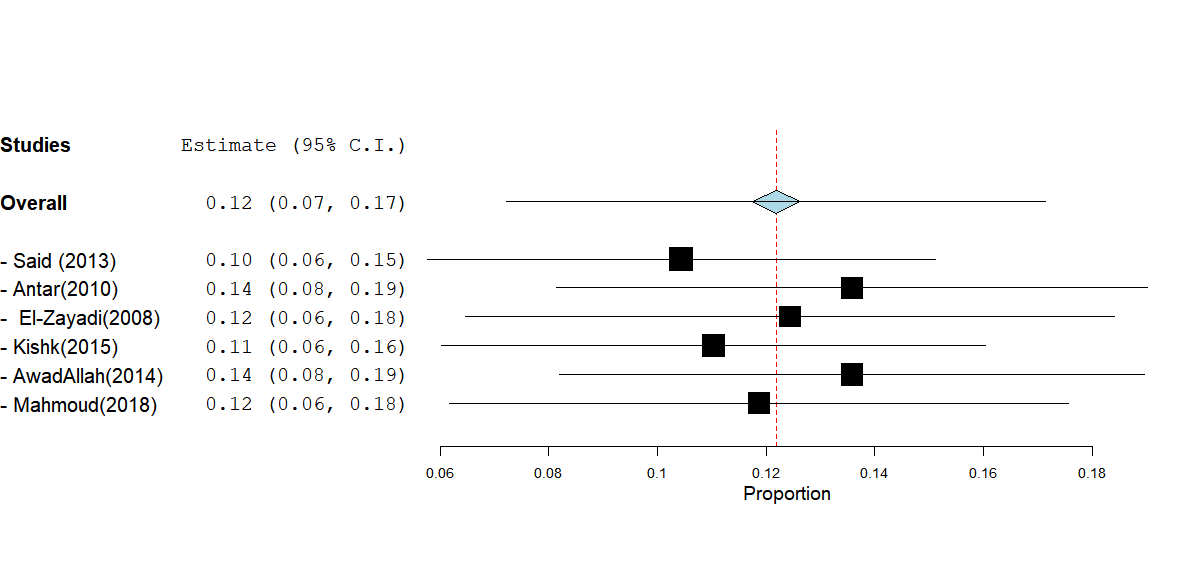


Sensitivity analysis of the pooled prevalence of occult hepatitis B infection in HBsAg negative and anti-HBc positive blood donors


**Fig.S8**


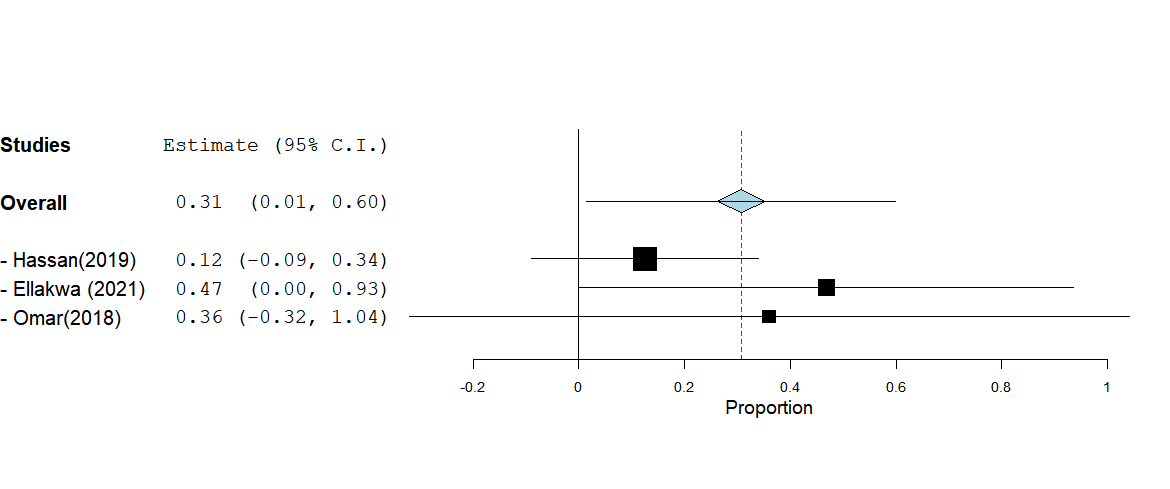

Sensitivity analysis of the pooled prevalence of occult hepatitis B infection in HBsAg negative and anti-HBc positive HCC patients


**Fig.S9**


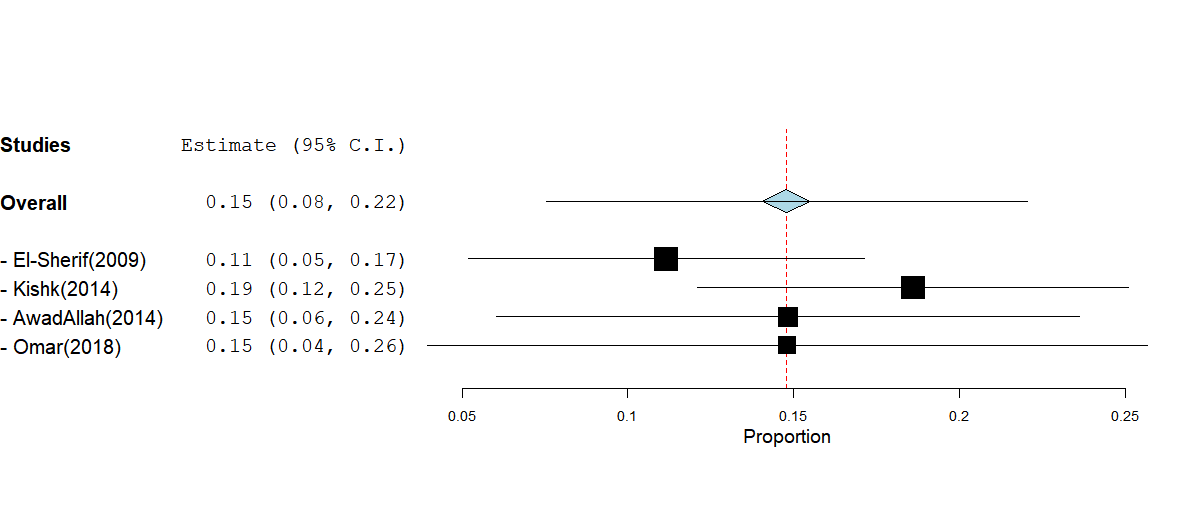

Sensitivity analysis of the pooled prevalence of occult hepatitis B infection in HBsAg negative and anti-HBc positive Chronic HCV infected patients


**Fig.S10**

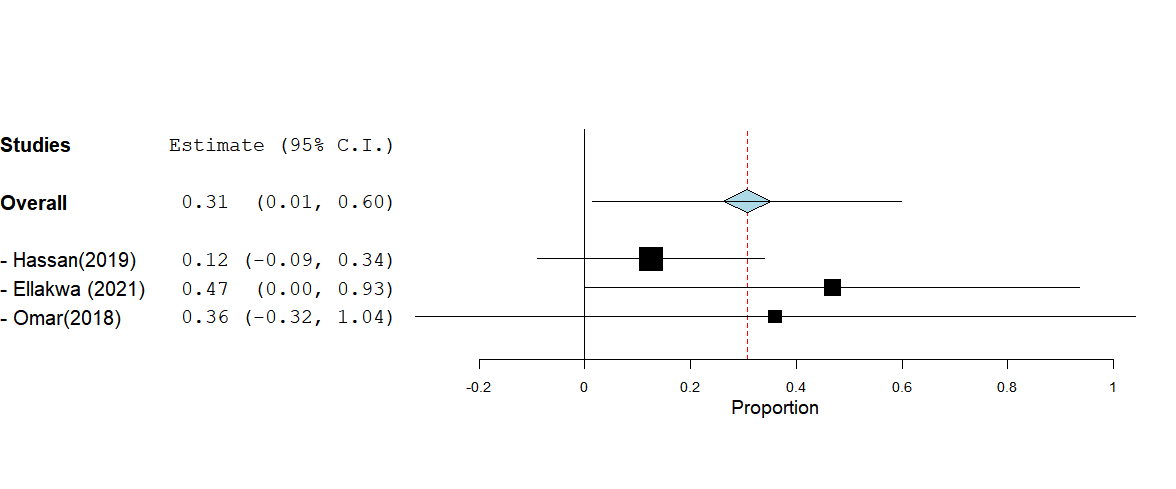


Sensitivity analysis of the pooled prevalence of occult hepatitis B infection in HBsAg negative and anti-HBc positive HCC patients

References
1. Ismail hisham, soliman M, Ismail nahed. Pathology and Laboratory Medicine International Dovepress Occult hepatitis B virus infection in egyptian hemodialysis patients with or without hepatitis c virus infection. *Pathol Lab Med Int* Published online 2010:2-113. Accessed November 12, 2022. https://www.dovepress.com/

2. Abu El Makarem MA, Hamid MA, Aleem AA, Ali A, Shatat M, Sayed D, Deaf A, Hamdy L, Tony EA. Prevalence of Occult Hepatitis B Virus Infection in Hemodialysis Patients From Egypt With or Without Hepatitis C Virus Infection. *Hepat Mon* 2012;12(4):253. doi:10.5812/HEPATMON.665

3. Saad El Dine S, El Said M, El-Dabaa E, Abdel Raoof E, Raafat M, Nabil M, Hassanein M, Saber M, Omer M. Manar Raafat, Malak Nabil, Moataz Hassanein, Mohamad Saber, Maisa Omer. Occult Hepatitis B Virus Infection in Haemodialysis Egyptian Patients with Chronic Hepatitis C. *Life Sci J* 2013;10(3):1097-8135. Accessed November 12, 2022. http://www.lifesciencesite.com.38

4. Esmail MA, Mahdi WKM, Khairy RM, Abdalla NH. Genotyping of occult hepatitis B virus infection in Egyptian hemodialysis patients without hepatitis C virus infection. *J Infect Public Health* 2016;9(4):452-57. doi:10.1016/J.JIPH.2015.11.018

5. Zaki MES, Rafaat D, Eliwa A, Abdelsalam M. Occult Hepatitis B among Patients under Hemodialysis at Mansoura University Hospitals: Prevalence and Risk Factors. *Virol Antivir Res*  2015;2014(01). doi:10.4172/2324-8955.1000118

6. Helaly GF, El Ghazzawi EF, Shawky SM, Farag FM. Occult hepatitis B virus infection among chronic hemodialysis patients in Alexandria, Egypt. *J Infect Public Health* 2015;8(6):562-69. doi:10.1016/J.JIPH.2015.04.019

7. Mohamed AS, Fattah NRA, Ismail WA, Abdel-Latif E. Occult Hepatitis B Virus Infection Among Hemodialysis Patients in Fakous General Hospital. *Egypt J Hosp Med* 2020;81(3):1648-53. doi:10.21608/EJHM.2020.117022

8. Mohamed NS, Abdelghani HM. High Prevalence of Occult Hepatitis B Virus Infection among Frequently Blood Transfused Children: A Single Egyptian Center Experience. *Int J Pediatr* 2020;8(12):12523-32. doi:10.22038/IJP.2020.52174.4147

9. Shaker O, Ahmed A, Satar IA, El Ahl H, Shousha W, Doss W. Occult hepatitis B in Egyptian thalassemic children. *J Infect Dev Ctries* 2011;6(04):340-46. doi:10.3855/jidc.1706

10. Said ZNA, El-Sayed MH, El-Bishbishi IA, El-Fouhil DF, Abdel-Rheem SE, El-Abedin MZ, Salama II. High prevalence of occult hepatitis B in hepatitis C-infected Egyptian children with haematological disorders and malignancies. *Liver Int* 2009;29(4):518-24. doi:10.1111/J.1478-3231.2009.01975.X

11. Gaber El Sheredy A, Abd El Kader Mahmoud O, Fathy El Ghazzawy E, Fahmy Helaly G, Aly El Naggar A, Muzamil Mahadi M. Occult Hepatitis B virus infection in patients with blood diseases. *IntJCurrMicrobiolAppSci* 2015;4(1):1-10. Accessed November 12, 2022. http://www.ijcmas.com

12. Emara MH, El-Gammal NE, Mohamed LA, Bahgat MM. Occult hepatitis B infection in egyptian chronic hepatitis C patients: Prevalence, impact on pegylated interferon/ribavirin therapy. *Virol J* 2010;7(1):1-8. doi:10.1186/1743-422X-7-324/TABLES/5

13. Omar HH, Taha SA, Hassan WH, Omar HH. Impact of schistosomiasis on increase incidence of occult hepatitis B in chronic hepatitis C patients in Egypt. *J Infect Public Health* 2017;10(6):761-65. doi:10.1016/J.JIPH.2016.11.010

14. El-Sherif A, Abou-Shady M, Abou-Zeid H, Elwassief A, Elbahrawy A, Ueda Y, Chiba T, Hosney AM. Antibody to hepatitis B core antigen as a screening test for occult hepatitis B virus infection in Egyptian chronic hepatitis C patients. *J Gastroenterol* 2009;44(4):359-64. doi:10.1007/S00535-009-0020-3/TABLES/3

15. El-Sherif WT, Sayed SK, Afifi NA, El-Amin HA, Wafaa [, El-Sherif T. Occult Hepatitis B Infection among Egyptian Chronic Hepatitis C Patients and its Relation with Liver Enzymes and Hepatitis B Markers. *Life Sci J* 2012;9(2):1097-8135. Accessed November 12, 2022. http://www.lifesciencesite.comhttp//www.lifesciencesite.comeditor@LifeScienceJournal.org467http://www.lifesciencesite.com.69.

16. Mahmoud OAEK, Ghazal AAER, Metwally DES, Shamseya MM, Hamdallah HM. Detection of occult hepatitis B virus among chronic hepatitis C patients. *Alexandria J Med* 2016;52(2):115-23. doi:10.1016/J.AJME.2015.06.003

17. Selim HS, Abou-Donia HA, Taha HA, El Azab GI, Bakry AF. Role of occult hepatitis B virus in chronic hepatitis C patients with flare of liver enzymes. *Eur J Intern Med* 2011;22(2):187-90. doi:10.1016/J.EJIM.2010.12.001

18. Naga M, Amin M, Algendy D, El Badry A, Fawzi M. Occult Hepatitis B Virus infection in a cohort of patients with chronic Hepatitis C. *Arch Hepat Res* 2019;5(1):017-21. doi:10.17352/AHR.000022

19. Thabit AG, Hassan MA, Agban MN, Makhlouf NA, Khalil NK, Hassan HAM, El-Mokhtar MA. Prevalence of Occult HBV Infection Among Chronic Hepatitis C Patients in Upper Egypt. The Egyptian journal of immunology. Published 2017. Accessed November 12, 2022. https://pubmed.ncbi.nlm.nih.gov/29120585/

20. EL-Bassuoni AM, Talaat MR, Fathy AA, Zamzam MS. Occult Hepatitis B and Carcinogenesis Markers in Chronic Hepatitis C Infection. *Egypt J Med Microbiol* 2014;23(2):75-84. doi:10.12816/0025686

21. El-Maraghy NN, Khalil FA, Dessouki O, Hassan R. Prevalence of occult HBV infection in HCV positive patients in Suez Canal area. *Int J Curr Microbiol Appl Sci* 2015;4(2):497-505. Accessed November 12, 2022. http://www.ijcmas.com/vol-4-2/Nermine%0AN.%0AEl-Maraghy,%0Aet%0Aal.pdf

22. Sheneef A, Yousef LM, Nor El-Din AK. Nor El-Din Occult Hepatitis B Infection in Patients with Chronic Hepatitis C. *Life Sci J* 2012;9(3):1097-8135. Accessed November 12, 2022. http://www.lifesciencesite.comhttp//www.lifesciencesite.com.41

23. Mandour M, Nemr N, Shehata A, Kishk R, Badran D, Hawass N. Occult HBV infection status among chronic hepatitis C and hemodialysis patients in Northeastern Egypt: regional and national overview. *Rev Soc Bras Med Trop* 2015;48(3):258-64. doi:10.1590/0037-8682-0037-2015

24. Hassan ZK, Hafez MM, Mansor TM, Zekri ARN. Occult HBV infection among Egyptian hepatocellular carcinoma patients. *Virol J* 2011;8(1):1-6. doi:10.1186/1743-422X-8-90/TABLES/4

25. Abd-Elfatah S, Salah Shabana H, M El-Kheshin GA. PREVALENCE OF OCCULT HBV INFECTION IN HEPATOCELLULAR CARCINOMA IN EGYPT. *AAMJ* 2013;11(4).

26. Daef EA, Makhlouf NA, Ahmed EH, Mohamed AI, Abd El Aziz MH, El-Mokhtar MA. Serological and Molecular Diagnosis of Occult Hepatitis B Virus Infection in Hepatitis C Chronic Liver Diseases. The Egyptian journal of immunology. Published 2017. Accessed November 12, 2022. https://pubmed.ncbi.nlm.nih.gov/29120576/

27. Taha SE, El-Hady SA, Ahmed TM, Ahmed IZ. Detection of occult HBV infection by nested PCR assay among chronic hepatitis C patients with and without hepatocellular carcinoma. *Egypt J Med Hum Genet* 2013;14(4):353-60. doi:10.1016/J.EJMHG.2013.06.001

28. El-Maksoud MA, Habeeb MR, Ghazy HF, Nomir MM, Elalfy H, Abed S, Zaki MES. Clinicopathological study of occult hepatitis B virus infection in hepatitis C virus-associated hepatocellular carcinoma. *Eur J Gastroenterol Hepatol* 2019;31(6):716-22. doi:10.1097/MEG.0000000000001388

29. Naeima KA, Eman AESM, Dina AR, Ola EMM. Occult hepatitis B virus among patients with chronic hepatitis and hepatocellular carcinoma. *Sci J Al-Azhar Med Fac Girls* 2018;2(3):205. doi:10.4103/SJAMF.SJAMF_36_18

30. Raouf HE, Yassin AS, Megahed SA, Ashour MS, Mansour TM. Seroprevalence of occult hepatitis B among Egyptian paediatric hepatitis C cancer patients. *J Viral Hepat* 2015;22(2):103-11. doi:10.1111/JVH.12260

31. El-Ghitany EM, Farghaly AG, Hashish MH. Occult hepatitis B virus infection among hepatitis C virus seropositive and seronegative blood donors in Alexandria, Egypt. *J Egypt Public Health Assoc* 2013;88(1):8-13. doi:10.1097/01.EPX.0000422774.29308.B3

32. Atef DM, Atef RM. Usefulness of nucleic acid testing among negative HBs Ag blood donors in Egypt. *Transfus Apher Sci* 2019;58(4):468-71. doi:10.1016/J.TRANSCI.2019.05.005

33. Azeem HA, Alkabeer AM, Mohammed AS, Fekry SG, Ch MBB. Prevalence of Occult Hepatitis B Virus Infection Among Assiut University Students. *Al-Azhar Int Med J* 2020;1(4):65-69. doi:10.21608/AIMJ.2020.22812.1098

34. Foaud H, Maklad S, Mahmoud F, El-Karaksy H. Occult hepatitis B virus infection in children born to HBsAg-positive mothers after neonatal passive-active immunoprophylaxis. *Infection* 2015;43(3):307-14. doi:10.1007/S15010-015-0733-6/TABLES/4

35. Elrashidy H, El-Didamony G, Elbahrawy A, Hashim A, Alashker A, Morsy MH, Elwassief A, Elmestikawy A, Abdallah AM, Mohammad AGS, Mostafa M, George NM, Abdelhafeez H. Absence of occult hepatitis B virus infection in sera of diabetic children and adolescents following hepatitis B vaccination. *http://dx.doi.org/104161/hv29521* 2014;10(8):2336-41. doi:10.4161/HV.29521

36. Said ZN, Sayed MHE, Salama II, Aboel-Magd EK, Mahmoud MH, Setouhy M El, Mouftah F, Azzab MB, Goubran H, Bassili A, Esmat GE. Occult hepatitis B virus infection among Egyptian blood donors. *World J Hepatol* 2013;5(2):64. doi:10.4254/WJH.V5.I2.64

37. Antar W, El-Shokry MH, Abd El Hamid WA, Helmy MF. Significance of detecting anti-HBc among Egyptian male blood donors negative for HBsAg*. *Transfus Med* 2010;20(6):409-13. doi:10.1111/J.1365-3148.2010.01021.X

38. El-Zayadi AR, Ibrahim EH, Badran HM, Saeid A, Moneib NA, Shemis MA, Abdel-Sattar RM, Ahmady AM, El-Nakeeb A. Anti-HBc screening in Egyptian blood donors reduces the risk of hepatitis B virus transmission. *Transfus Med* 2008;18(1):55-61. doi:10.1111/J.1365-3148.2007.00806.X

39. Mahmoud AI, Elsherbiny NM, Afifi NA, Ahmed BM, Yasin AS. Occult Hepatitis B Infection Among Blood Donors in Al Azhar University Hospital, Upper Egypt: The Current Status After 25 years of Vaccine Introduction. *Egypt J Immunol* 2018;25(1):45-56. Accessed November 17, 2022. https://europepmc.org/article/med/30242997

40. Kishk R, Nemr N, Elkady A, Mandour M, Aboelmagd M, Ramsis N, Hassan M, Soliman N, Iijima S, Murakami S, Tanaka Y, Ragheb M. Hepatitis B surface gene variants isolated from blood donors with overt and occult HBV infection in north eastern Egypt. *Virol J* 2015;12(1):1-8. doi:10.1186/S12985-015-0389-Y/TABLES/3

41. Abdelaziz NF, Fekry MM, Hashish MH. Occult Hepatitis B Virus Infection in Egyptian HIV-Infected Patients with Isolated Anti-HBc. *J High Inst Public Heal* 2019;49(3):162-67. doi:10.21608/JHIPH.2019.59201

42. Elmaghloub R, Elbahrawy A, El Didamony G, Hashim A, Morsy MH, Hantour O, Hantour A, Abdelbaseer M. Occult hepatitis B infection in Egyptian health care workers. *East Mediterr Health J* 2017;23(5):329-34. doi:10.26719/2017.23.5.329

43. R. K, H. AA, M. R, M. K, L. M, Kishk R, Atta HA, Ragheb M, Kamel M, Metwally L, Nemr N. Genotype characterization of occult hepatitis B virus strains among Egyptian chronic hepatitis C patients. Eastern Mediterranean Health Journal. Published 2014. Accessed November 12, 2022. http://www.emro.who.int/emhj-vol-20-2014/volume-20-issue-2/genotype-characterization-of-occult-hepatitis-b-virus-strains-among-egyptian-chronic-hepatitis-c-patients.html%0Ahttp://applications.emro.who.int/emhj/v20/02/EMHJ_2014_20_2_130_138.pdf%0Ahttp://ov

44. AWadAllah AA, Farahat MH, Amr GE, El-Sheikh AR, Bahgat MM. Occult Hepatitis B Infection in Blood Donors at Zagazig University Hospitals , Sharkia Governorate. The Egyptian Journal of Medical Microbiology. doi:10.12816/0025684

45. Omar H, Taha S, Hassan W, Omar H. Occult hepatitis B infection: a hidden factor of poor response to intervention treatment of hepatocellular carcinoma in chronic hepatitis C patients. *Comp Clin Path* 2018;27(5):1273-79. doi:10.1007/S00580-018-2735-0/TABLES/4

46. Hassan MS, Abdelmalek MO, Youssif LM, Hassanein SAAE. Occult hepatitis B virus infection in patients with hepatitis C virus-related cirrhosis with or without hepatocellular carcinoma. *J Curr Med Res Pract* 2019;4(3):308-13. doi:10.4103/JCMRP.JCMRP_58_18

47. El-Sayed Ellakwa D, Abdel-Hamid M, Seif EL-Din Ashour M, Sayed Khairy L El, Ali OSM. Identifying of HBV DNA in liver tissues of chronic hepatitis and hepatocellular carcinoma to study the Hepatitis B virus silent infection in Egyptian patients. *Ecol Genet Genomics* 2021;18:100077. doi:10.1016/J.EGG.2020.100077

48. Berbesh A. OCCULT HEPATITIS B INFECTION IN HEMODIALYSIS PATIENTS INFECTED WITH HEPATITIS C VIRUS. *Al-Azhar Med J* 2021;50(2):1553-62. doi:10.21608/AMJ.2021.160386

49. Elbedewy TA, Elshweikh Samah A, Baiomy Nivin. Prevalence and significance of hepatitis-B core antibodies among hepatitis B surface antigen-negative Egyptian patients on hemodialysis in Al-Gharbia governorate. *Tanta Med J* 2016;44(2):33-38.

50. Elkady A, Iijima S, Aboulfotuh S, Ali EM, Sayed D, Abdel-Aziz NM, Ali AM, Murakami S, Isogawa M, Tanaka Y. Characteristics of escape mutations from occult hepatitis B virus infected patients with hematological malignancies in South Egypt. *World J Hepatol* 2017;9(9):477. doi:10.4254/WJH.V9.I9.477

51. Naing L, Winn T, Rusli BN. Practical Issues in Calculating the Sample Size for Prevalence Studies. *Arch Orofac Sci* 2006;1:9-14. Accessed November 18, 2022.
